# Surgical pleth index to predict the success of caudal block in pediatric patients under general anesthesia

**DOI:** 10.1186/s12871-025-03353-y

**Published:** 2025-10-14

**Authors:** Ruchita Harchandani, Deepali Shetty, Anitha Nileshwar, Handattu Mahabaleswara Krishna, Suvajit Podder

**Affiliations:** 1 Department of Anesthesiology, Kasturba Medical College, Manipal, Manipal Academy of Higher Education, Manipal, Karnataka State 576104 India; 2 Department of Anesthesiology, All India Institute of Medical Sciences, Mangalagiri, 522503 Andhra Pradesh India

**Keywords:** Analgesia, Caudal, Children, Nociception, Surgical pleth index

## Abstract

**Background:**

Caudal block is usually performed after general anesthesia in children, which makes it hard to assess the success of the block. The aim of this study was to determine whether the surgical pleth index can serve as an objective tool for assessing nociception and the success of caudal block in anesthetized children.

**Methodology:**

Sixty-two children aged 1–6 years, with American Society of Anesthesiologists Physical Status I and II, undergoing elective infraumbilical surgery, were included. All patients received general anesthesia with i-gel and caudal analgesia. The surgical pleth index, heart rate, and mean blood pressure were recorded throughout the procedure. Caudal block was assessed postoperatively with a gentle pinch.

**Results:**

The patients were divided into Group S (caudal success) and Group F (caudal failure). In both groups, the SPI decreased after induction to nearly < 50. It continued to gradually decrease to ≈30 in Group S, whereas it increased sharply with incision in Group F to ≈76. After additional analgesia with fentanyl, the surgical pleth index decreased in Group F, but the mean surgical pleth index was significantly greater than in Group S (*p* < 0.01). The intergroup difference in surgical pleth index persisted throughout the procedure. A receiver operating curve showed that a surgical pleth index of 43.5 before incision has a sensitivity of 83.3% and a specificity of 67.9% for predicting successful caudal block. Heart rate and blood pressure increased significantly with incision in Group F, unlike in Group S. The intergroup difference in hemodynamics disappeared after additional analgesia.

**Conclusion:**

A surgical pleth index of ≤ 43.5 (which may be rounded to 40) can serve as a nociceptive index to predict the success of a caudal block in children.

**IEC registration number:**

IEC2 549/2023 dated 19th March 2024.

**Clinical trials registration:**

(CTRI/2024/12/078281) dated 18th December 2024.

## Background

Effective pain management is crucial for patient well-being and enhancing surgical outcomes. It should be established before the surgical incision is made and continue throughout surgery and into the postoperative period. Caudal block is a regional anesthesia technique commonly used in pediatric patients to provide adequate analgesia for subumbilical abdominal surgeries, genitourinary procedures, and lower limb surgeries [1]. It is typically administered after general anesthesia in children, which makes assessing the success of the block challenging.

Traditionally, hemodynamic changes such as an increase in heart rate or blood pressure are used to predict the success of a caudal block, but these factors are considered unreliable [2]. Several tests, like ‘swoosh’ and ‘whoosh’ tests and ultrasound, can be employed to ensure correct needle placement and increase the likelihood of block success [3, 4]. Various other parameters, such as laxity of the anal sphincter tone and loss of the cremasteric reflex, are also used; however, the latter can only be applied to male children, and their reliability is questionable under general anesthesia [5]. Therefore, an objective parameter is needed to assess the success of the block.

The surgical plethysmographic index (SPI) is a technique that objectively evaluates analgesia [6]. It accurately measures nociception levels during surgery under general anesthesia, providing improved guidance for administering different analgesics [7]. Painful stimuli trigger a sympathetic response within the autonomic nervous system. The SPI calculates these responses from the plethysmographic signal recorded by a pulse oximeter. It provides real-time feedback to help anesthesiologists adjust anesthesia and analgesia levels, ultimately enhancing patient comfort and safety during surgery [8].

SPI is calculated according to the formula: SPI = 100 – (0.33 x HBI + 0.67 x PPGA), where HBI is the heartbeat interval and PPGA is the plethysmographic waveform amplitude. This formula takes into account the systolic pressure and photoplethysmographic waveform amplitude to calculate the heartbeat index (HBI). Thus, the SPI decreases if HBI increases (lower heart rate) or the PPGA amplitude increases (good pulse and vasodilatation). The values range from 0 to 100. Studies on SPI suggest that values of 20–50 indicate good nociception in adults, but its utility in children is not well established [9–13]. 

In this study, the primary objective was to evaluate whether the SPI can serve as an objective nociceptive tool for assessing the success of a caudal block in anesthetized children. The secondary objective was to determine how accurately the anesthesiologist could diagnose the success of the caudal block intraoperatively.

## Methodology

This prospective observational study was conducted in accordance with the principles of the Declaration of Helsinki. Approval was obtained from the Ethics Committee of the University—IEC Registration number: IEC2 549/2023, dated March 19, 2024, and Clinical Trials Registration: (CTRI/2024/12/078281), dated December 18, 2024.

Children aged 1 to 6 years with ASA Physical Status Class I or II, undergoing elective infraumbilical surgery under general anesthesia using a supraglottic airway device and caudal analgesia, were included. Patients with preexisting liver, renal, or cardiac disorders, developmental delays, known organic brain disease, cerebral palsy, or contraindications to caudal analgesia were excluded.

The patient was assessed for enrollment the day before surgery. After enrollment, written informed consent was obtained from the patient’s parents. The patient was advised to avoid solid food for at least 6 h, breast milk for 4 h, and clear fluids for 2 h before the procedure.

There were three observers in the study: Observer 1 was a postgraduate in anesthesia and the principal investigator, responsible for screening patients preoperatively to ensure they met the inclusion criteria and did not have any of the exclusion criteria. Observer 2 was a consultant anesthetist who administered the anesthesia. Observer 3 was a postgraduate in anesthesiology who was unaware of whether the caudal epidural was working. Observer 3 assessed the analgesia and the caudal site postoperatively.

All patients had an intravenous cannula placed in parental presence by the paediatrician or paediatric surgeon on the day before surgery. The same cannula was used to draw a blood sample for preoperative investigations. All patients received intravenous (IV) midazolam (0.05–0.1 mg/kg) in the preoperative holding area. Standard monitoring included pulse oximetry, noninvasive blood pressure (BP), electrocardiogram (ECG) monitoring (Lead II and V5), capnography, and volatile anesthetic monitoring (Carescape B650 multimodular monitor from GE, Finland).

Anesthesia was induced with IV fentanyl (0.5 mcg/kg) and IV propofol (2–3 mg/kg) until loss of consciousness. This was followed by the insertion of a supraglottic airway device. Anesthesia was maintained with oxygen, air, and isoflurane titrated to a minimum alveolar concentration of 1. The patient was then positioned in the lateral decubitus position and given a caudal block. Under strict aseptic precautions, a caudal epidural needle was inserted, and 1 mL/kg of 0.25% bupivacaine was administered. The patient was then moved to a supine position. After the caudal block, the SPI, heart rate (HR), and mean arterial pressure (MAP) were recorded. The surgical incision was made approximately 10 min after the caudal block was administered.

SPI was monitored alongside HR and BP throughout the procedure as an additional parameter. It was recorded at baseline, just before caudal, and every 5 min until skin closure began. The anesthesiologist administered a rescue dose of IV fentanyl (0.5–2 mcg/kg) if they believed the block to be ineffective based on clinical judgment.

Postoperatively, 30 min after surgery, an anesthesiologist, who was blinded to the intraoperative SPI or the course of anesthesia throughout surgery, assessed the success of the caudal block when the child was calm or sleeping. A gentle pinch was applied to the skin starting from the distal part of both lower limbs, and the patients were observed for signs of grimacing, wincing, withdrawal, or changes in heart rate. If no response was observed, the stimulus was progressively applied more proximally until the expected level of blockade was reached (inguinal region). The verbal response of a communicating child was used to assess the success of the block.

When examined postoperatively, six of the patients responded to a skin pinch, while 56 did not respond. Based on the response, the children were divided into two groups: Group S, where the caudal block was successful, and Group F, where the caudal block appeared to have failed.

Sample size estimation and statistical analysis: The study’s sample size for the study was based on pilot data on 10 patients, according to which the proportion of subjects with diagnostic accuracy of the SPI was 80%. The formula provided by Lemeshow et al. was used [14]:

$$\mathrm{Sample}\;\mathrm{size}\;\mathrm N=\frac{\left(z_{1-\left(\alpha\:/2\right)}\right)^2\times\:\:p\:\times\:\:\left(1-p\right)}{\delta^2}$$

Proportion of subjects with diagnostic accuracy of the SPI: *p* = 0.80 (80%); precision: δ = 0.10 (10%); type I error: α = 0.05 (5%), $\:z_{\left(1-\alpha\:/2\right)}=1.96$; power: 80%.

Based on the formula and values given above:

The sample size required was N = [1.96^2^ × 0.80 × (1-0.80)]/0.10^2^ = 61.46 ≈ 62. Thus, with a 95% confidence interval, the proposed sample size for the study was 62.

Means/standard deviations and medians/interquartile range (IQR) for continuous variables and frequencies and percentages for categorical data were used. The normality of the continuous data was checked using the Shapiro‒Wilk test. Statistical significance was set at *p* < 0.05. Friedman’s test for intra- and intergroup comparisons and the Chi-square test were used for categorical data.

## Results

**Fig. 1:**
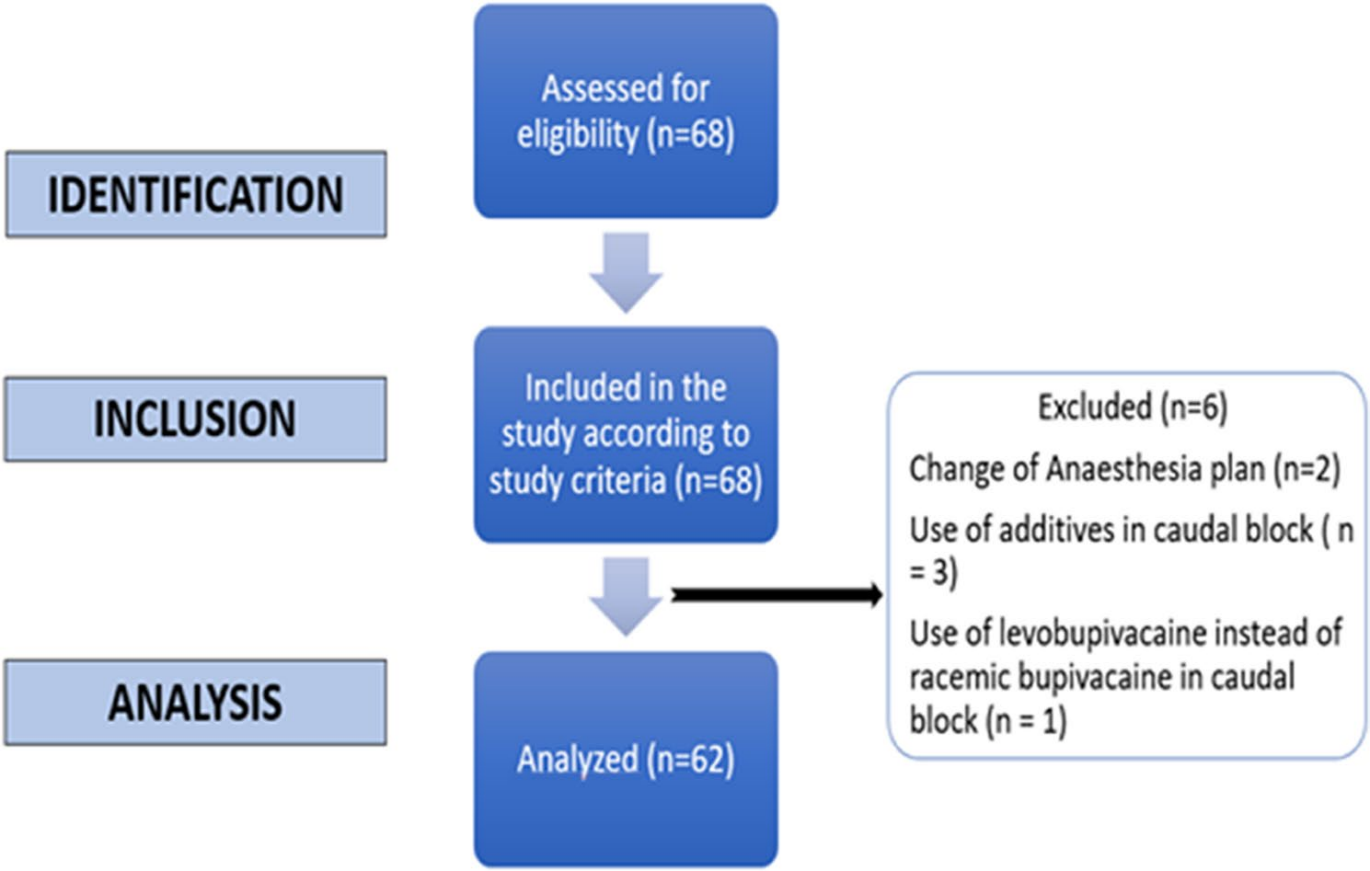
STROBE Flowchart

We included a total of 68 patients scheduled for surgery under general anesthesia with a supraglottic airway device, and caudal block (Fig. 1). Two patients were excluded due to a change in the anesthesia plan from a supraglottic airway device to endotracheal intubation. Three patients were excluded because of the use of additives in the caudal block due to the longer expected surgical time. One patient was excluded because of the use of levobupivacaine instead of bupivacaine in the caudal block. Data from 62 patients were analyzed.

Table 1 presents the demographic details of the study participants. Children were scheduled for surgeries such as herniotomy, circumcision, orchidopexy, hypospadias repair, or orthopedic procedures.

**Table 1:** Demographic details (*n* = 62)

Age (years) (Median [IQR] [Range]	2 [1, 4] [1–6]
Weight (kg) (Mean ± SD)	12.94 ± 3.38
Height (cm) (Mean ± SD)	93.7 ± 14.2
Gender M/F [*n* (%)]	55/7 (88.7/12.3)
ASA PS (I/II) [*n* (%)]	59/3 (95.16/4.84)
Duration of surgery (min) Median [IQR] (Range)	40 [30–60] (20–120)

*IQR* Interquartile range; *M* Male; *F* Female.; *ASA PS* American Society of Anesthesiologists Physical status

**Fig. 2:**
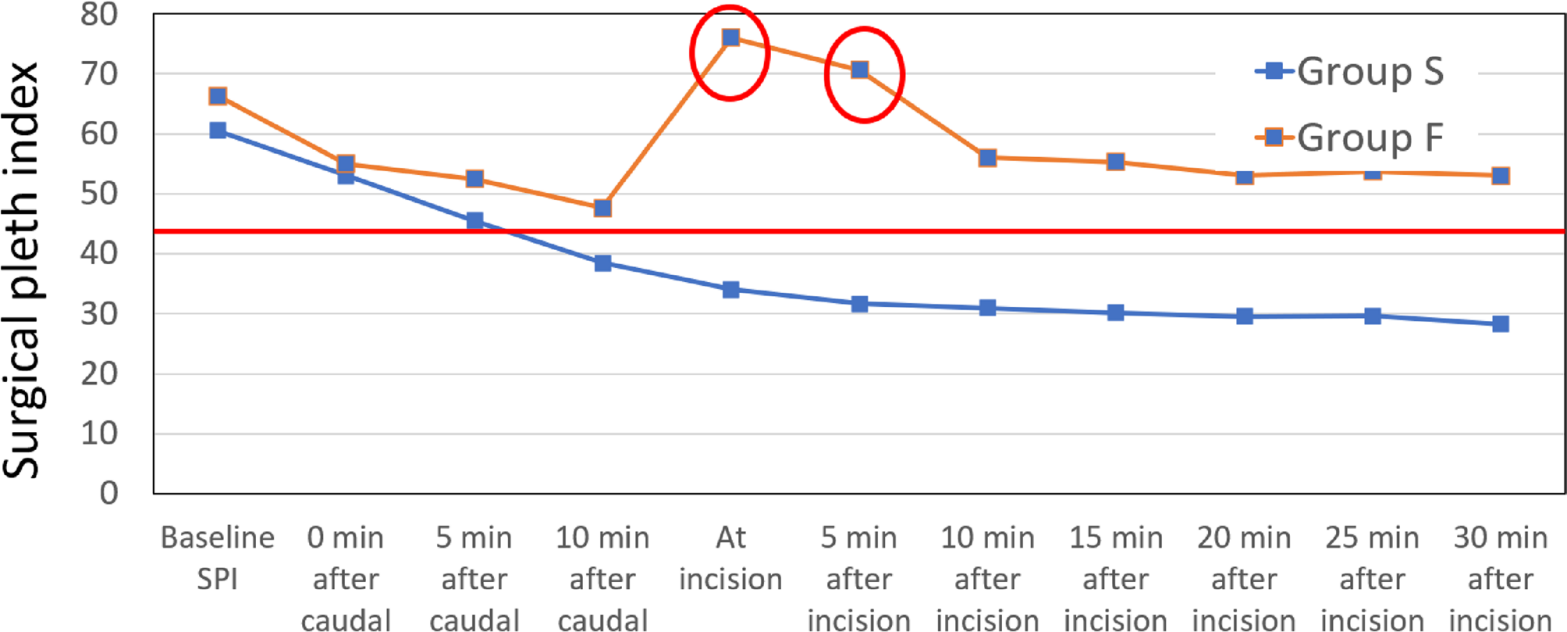
Mean SPI at baseline, after the block, and after the incision. The circled points on the line indicating failed caudal blocks highlight the sharp increase in SPI at the incision. The red horizontal line indicates the discriminatory SPI value of 43.5

The patterns of SPI, heart rate, and blood pressure changes after caudal and upon incision in these patients were analyzed to identify any differences between the two groups. Changes in the surgical pleth index (SPI) were compared at various time points: baseline, after the caudal block, and after skin incision at 5-minute intervals (Fig. 2). Intragroup comparison of all parameters was done with the baseline reading of the respective parameters that were assessed.

**Fig. 3:**
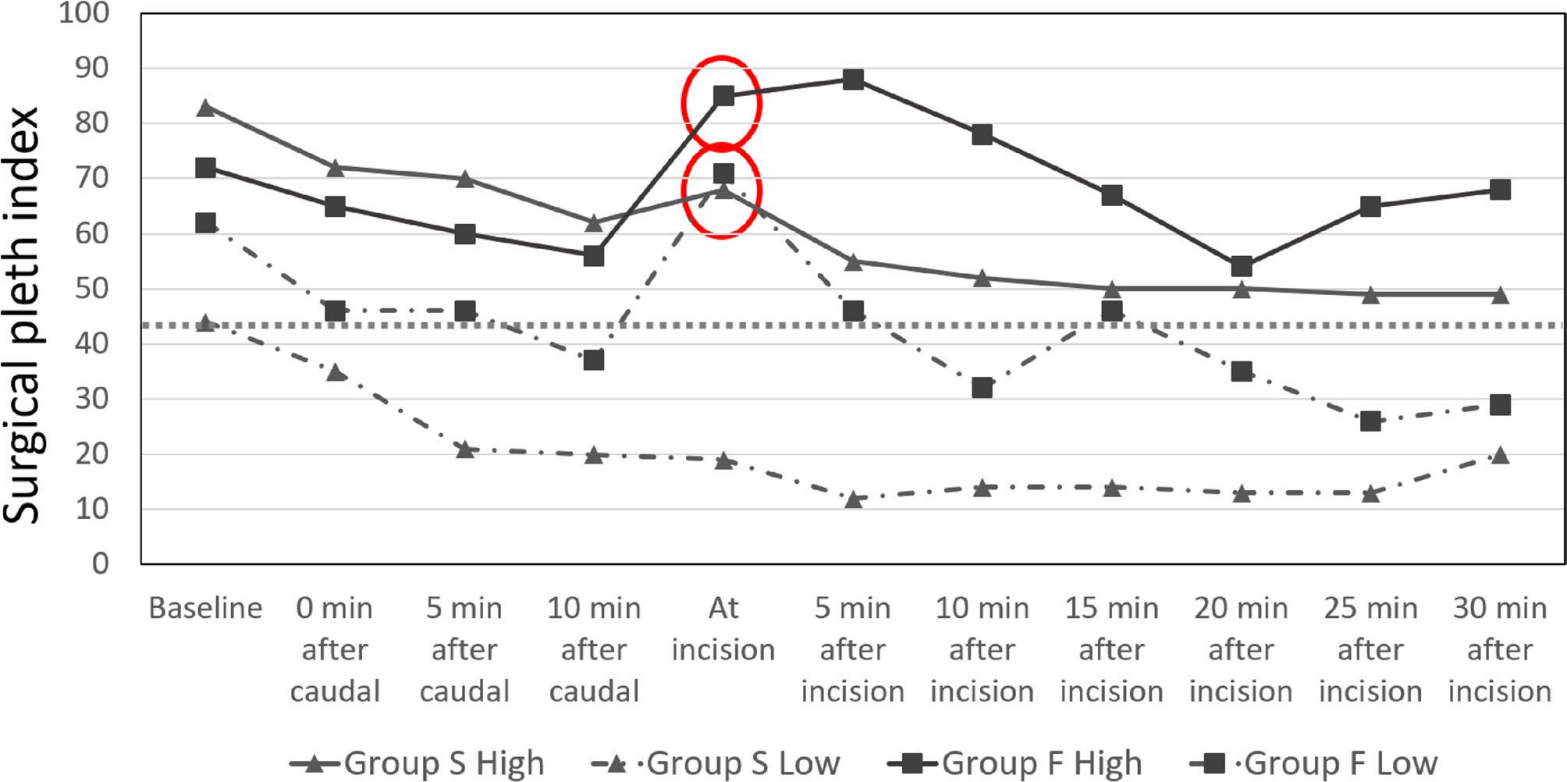
Highest and lowest SPI at baseline, after the block and after incision. The dotted line represents a SPI of 43.5

The SPI decreased after the induction of anesthesia in both groups. It further declined in patients with successful caudal blocks and continued to drop over time. In group F, however, the SPI sharply increased to approximately 76 when the surgical incision was made 10 min after induction. After administering fentanyl, the SPI gradually decreased, but the mean SPI remained higher in the failed caudal group than in the successful caudal group.The highest and lowest values of SPI seen in the successful and failed caudal groups at various time points are shown in Fig. 3.

The Friedman test was used to analyze the two groups. There was a significant intergroup difference at 10 min after caudal injection, which became highly significant thereafter (*p* < 0.01). The intragroup comparison also showed a highly significant difference in Group S (< 0.01), while there was no intragroup difference in Group F (*p* = 0.057).

**Fig. 4:**
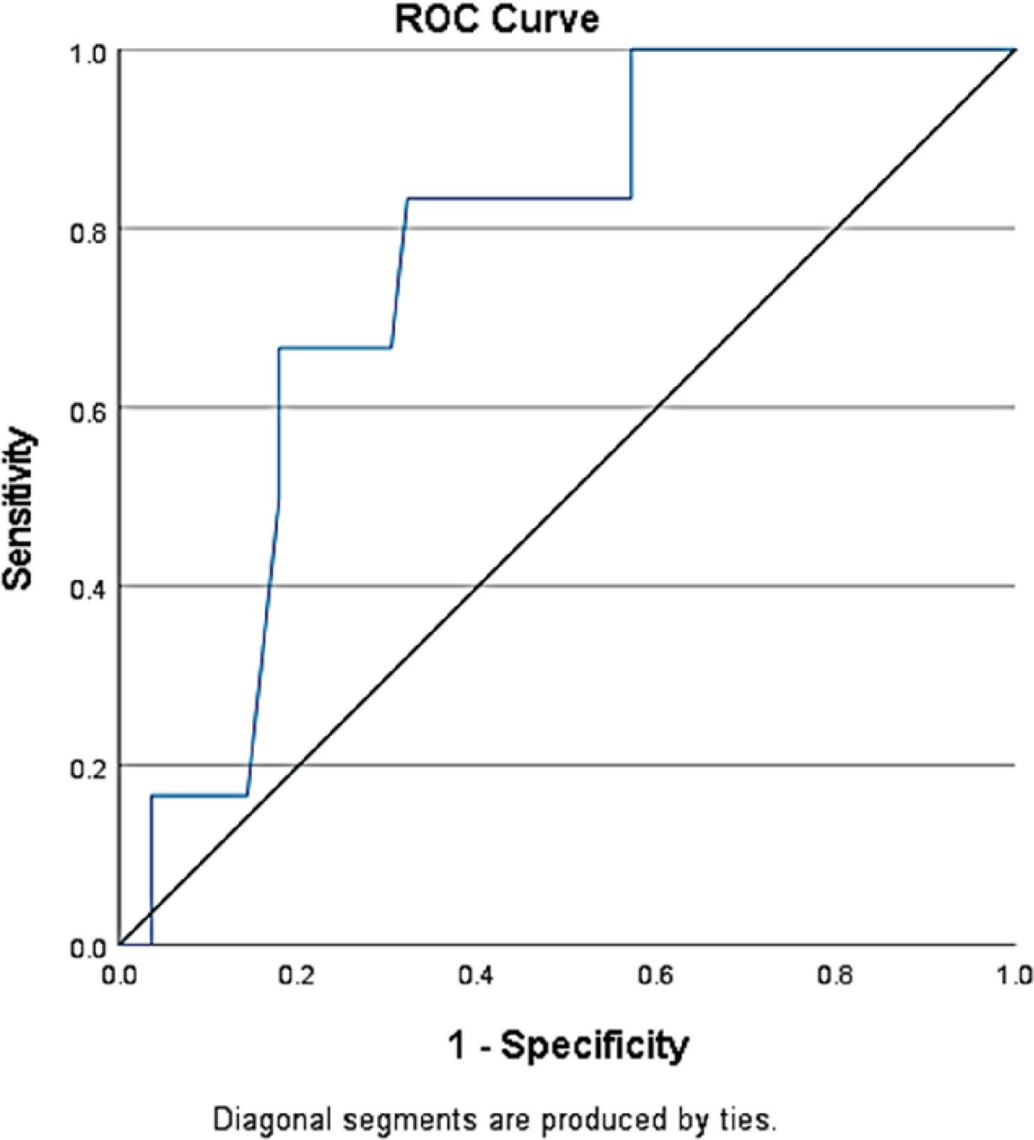
Receiver operating characteristic (ROC) curve for SPI values prior to incision

 Ledowski et al. [15] mentioned an SPI of 30 for differentiating between adequate and inadequate analgesia during general anesthesia. In our study, a receiver-operating curve was created using SPI values from the successful and failed caudal groups (Fig. 4). A value of 43.5 was obtained, with 83.3% sensitivity and 67.9% specificity in predicting the success of the caudal block.

**Fig. 5:**
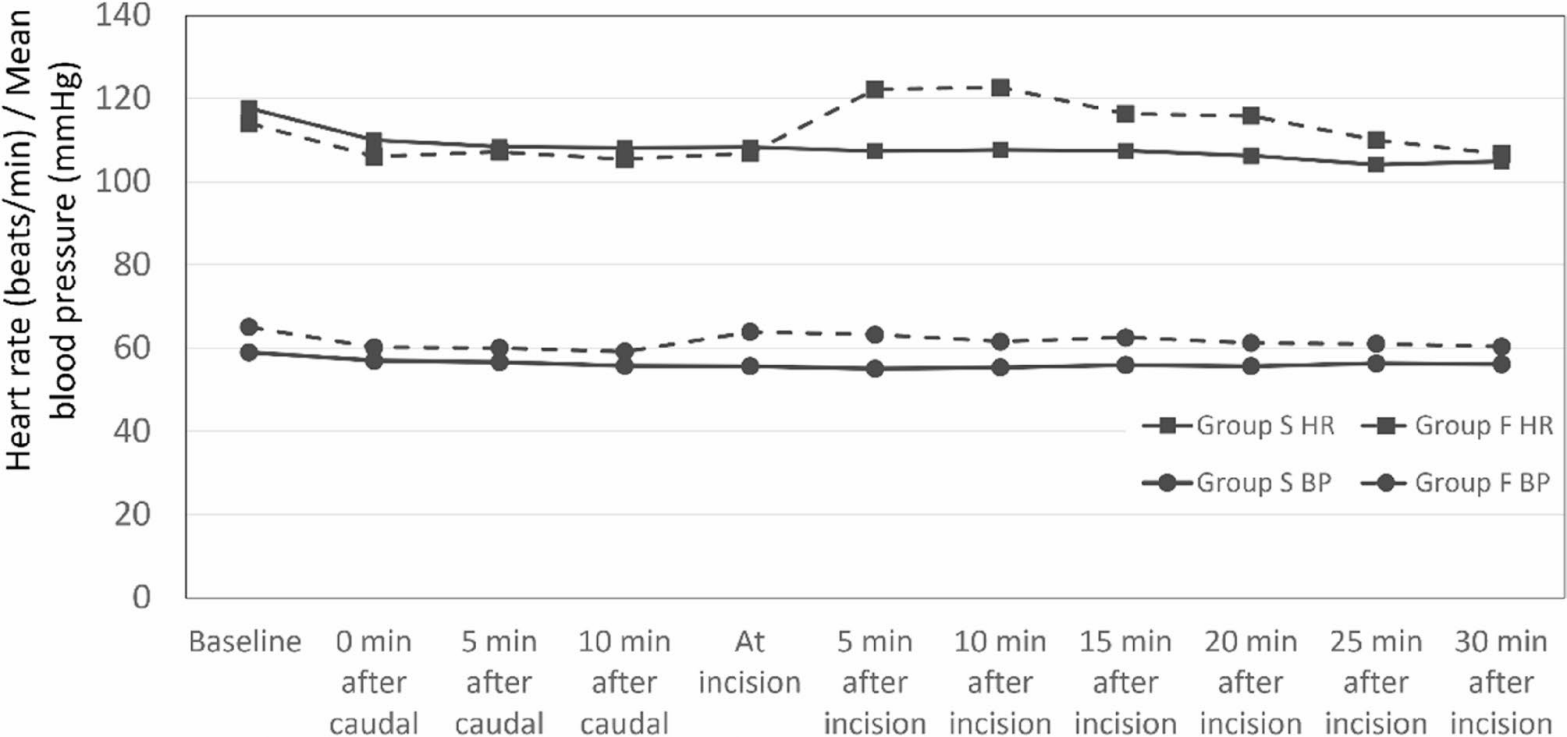
Heart rate and mean blood pressure at baseline, after the block, and after the incision

The changes in heart rate from baseline, after caudal, and after incision are shown in Fig. 3. As expected, the heart rate increased significantly with incision in patients in whom the caudal block failed, while there was no change in those with a successful caudal block. The intergroup difference was significant at the time of incision and for up to 10 min afterward. Patients in the failed caudal group received additional analgesia, and there was no difference between groups thereafter. Changes in blood pressure from baseline, after caudal block, and after incision, which mirrored those of heart rate, are also displayed in Fig. 5. The Friedman test was used to analyze these changes, revealing an intragroup P value of < 0.01 for Group S and < 0.01 for Group F.

The intraoperative assessment of the success of the caudal block by the anesthetist was 100% accurate, as confirmed by the postoperative pinch test (where there was no response in 56 patients but in 6 patients who moved with the pinch). All six patients diagnosed with a failed caudal block had required rescue analgesia (fentanyl) intraoperatively.

## Discussion

Several studies have demonstrated the usefulness of the surgical pleth index in predicting the adequacy of analgesia during surgery and the postoperative period [9–11, 15–20]. A few studies have highlighted the challenges of applying adult SPI thresholds (20–50) in pediatric patients due to their immature autonomic nervous systems and different vascular physiology [12, 13, 21, 22]. This study aimed to assess the reliability of the surgical pleth index (SPI) in predicting the success of caudal blocks in pediatric patients under general anesthesia. The results are especially significant because there is limited evidence supporting the use of SPI in pediatric populations.

Our findings showed that the SPI values decreased in all patients after induction of anesthesia, dropping from preinduction levels of about 60–65 to around 50. At ten minutes after the caudal block was placed, the difference in SPI between the groups was not significant. In patients with successful caudal anesthesia, the SPI values continued to decrease further, initially reaching 30 and then dropping to 20, which was sustained even after incision.

In patients where the caudal block was unsuccessful, the SPI values rose significantly to 70 with the incision and continued to remain high. There were four patients in Group S (successful caudal), where, although the block was successful, with incision, the SPI was greater than 50, despite unchanged hemodynamics. This was a mild increase in SPI and unlike in the failed caudal group, decreased to less than 50 in the next five min and to lower levels thereafter. None of these patients needed further analgesia. Perhaps the onset of caudal analgesia was delayed in these four patients, but it eventually took effect, as evidenced by the absence of response to the pinch postoperatively. Since the patient was also under general anesthesia, it would not have been appropriate or practical to wait more than ten minutes after the caudal to proceed with incision and surgery.

The consultant anesthetist responsible for the patient had access to the SPI values but made decisions solely based on clinical judgment and changes in heart rate and blood pressure, rather than the SPI. The protocol allowed the anesthetist to administer additional doses of fentanyl if they believed the caudal block was ineffective or if the patient needed additional analgesia intraoperatively. None of the patients in the successful caudal group received additional fentanyl, while all six patients in the failed group required it. After fentanyl was given and pain was better controlled, the SPI dropped to 50 in the failed group but did not decrease further, unlike in the successful group.

The intergroup difference (between Group S and Group F) in SPI trends was statistically significant, supporting the potential of SPI as a reliable nociceptive index. This finding aligns with studies by Huiku et al. [5] and Ledowski et al. [10], which demonstrated that SPI can detect nociceptive responses under general anesthesia. The ROC curve in our study indicated that a value of 43.5 effectively differentiates between successful and failed caudal blocks, with 83.3% sensitivity and 67.9% specificity. For simplicity, this number can be rounded to 40. Ledowski et al. [10] proposed a target SPI value of 40 but reported a sensitivity of 76%, a specificity of 62%, and a negative predictive value of 87.5%. Park et al. used a target value of 50. In their study, fentanyl requirements were lower in the SPI-guided group, but emergence agitation was higher. They stated that SPI does not appear to be valid in children [12].

The difference in the SPI between the failed and successful caudal groups persisted even up to 30 min after incision. Changes in heart rate and blood pressure were significant only during the first 15 min after incision. We examined how accurately anesthetists predict the success of caudal blocks and their response to nociceptive stimuli. No discrepancy was found between the anesthesiologist’s intraoperative impression and the postoperative assessment via the pinch test.

Factors such as probe placement, peripheral perfusion, preoperative anxiety, and movement during surgery can influence SPI readings. Future research could investigate the use of multimodal nociceptive indices, like combining the SPI with the analgesia nociception index (ANI) or nociception level index (NoL), to enhance predictive accuracy.

## Limitations

This study has certain limitations. The small sample size, especially for the failed caudal block group, may affect the generalizability of the results. SPI values can be influenced by artifacts, vasopressors, and autonomic variability, although our protocol aimed to minimize these confounders. Future studies with larger samples and multicenter designs are needed to confirm these findings and refine age-specific SPI thresholds.

In conclusion, a successful caudal block is associated with little or no change in the surgical pleth index (SPI), while failure causes a significant increase in SPI immediately after incision. SPI ≤ 40 can serve as a nociceptive index to predict the success of caudal block in children. Additionally, SPI decreases more with caudal analgesia than with intravenous analgesia.

## Data Availability

Data is with the corresponding author and can be made available on request.

## Declarations

### Use of any animal or human data or tissue

“Not applicable”.

## Abbreviations

SPI: Surgical pleth index
OR: Operating room
IV: Intravenous
HR: Heart rate
BP: Blood pressure
IQR: Interquartile range
ROC curve: Receiver operating characteristic curve
